# Self-reported physical activity and objective aerobic fitness: differential associations with gray matter density in healthy aging

**DOI:** 10.3389/fnagi.2015.00005

**Published:** 2015-02-03

**Authors:** Zvinka Z. Zlatar, Keith M. McGregor, Stephen Towler, Joe R. Nocera, Joseph M. Dzierzewski, Bruce Crosson

**Affiliations:** ^1^Department of Psychiatry, Stein Institute for Research on Aging, University of CaliforniaSan Diego, La Jolla, CA, USA; ^2^Department of Neurology, Emory UniversityAtlanta, GA, USA; ^3^Center for Visual and Neurocognitive Rehabilitation, Atlanta VA Medical CenterDecatur, GA, USA; ^4^Geriatric Research, Education and Clinical Center, VA Greater Los Angeles Healthcare SystemLos Angeles, CA, USA; ^5^David Geffen School of Medicine, University of California, Los AngelesLos Angeles, CA, USA; ^6^Department of Psychology, Georgia State UniversityAtlanta, GA, USA

**Keywords:** physical activity, aerobic fitness, voxel based morphometry, healthy aging, MRI, gray matter density

## Abstract

Aerobic fitness (AF) and self-reported physical activity (srPA) do not represent the same construct. However, many exercise and brain aging studies interchangeably use AF and srPA measures, which may be problematic with regards to how these metrics are associated with brain outcomes, such as morphology. If AF and PA measures captured the same phenomena, regional brain volumes associated with these measures should directly overlap. This study employed the general linear model to examine the differential association between objectively-measured AF (treadmill assessment) and srPA (questionnaire) with gray matter density (GMd) in 29 cognitively unimpaired community-dwelling older adults using voxel based morphometry. The results show significant regional variance in terms of GMd when comparing AF and srPA as predictors. Higher AF was associated with greater GMd in the cerebellum only, while srPA displayed positive associations with GMd in occipito-temporal, left perisylvian, and frontal regions after correcting for age. Importantly, only AF level, and not srPA, modified the relationship between age and GMd, such that higher levels of AF were associated with increased GMd in older age, while decreased GMd was seen in those with lower AF as a function of age. These results support existing literature suggesting that both AF and PA exert beneficial effects on GMd, but only AF served as a buffer against age-related GMd loss. Furthermore, these results highlight the need for use of objective PA measurement and comparability of tools across studies, since results vary dependent upon the measures used and whether these are objective or subjective in nature.

## Introduction

A strong link has been established between higher physical activity (PA) levels and improved cognitive function in aging. Epidemiological studies have found that higher midlife PA is associated with lower risk of cognitive decline and dementia later in life (Hamer and Chida, 2009; Sofi et al., 2011), while exercise intervention studies have identified changes in brain structure and function that are related to improved cognition in the elderly (for a review, see Hayes et al., 2013). Mechanisms by which exercise affects cognitive function in humans include cell proliferation and increased synaptic density (Pereira et al., 2007), angiogenesis (Swain et al., 2003), changes in mitochondrial function (Steib et al., 2014), and alteration of trophic factor signaling, which in turn affects neuronal function and structure in areas that are critical for cognitive function (Phillips et al., 2014). One such example are exercise-induced increases in gene expression of brain derived neurotrophic factor (BDNF), which is critical for learning and memory formation by providing a propitious environment for neuroplasticity. Moreover, exercise has been shown to confer vascular, immunological, and anti-inflammatory benefits as well as changes in brain function, connectivity and perfusion (for reviews of the literature, see Voss et al., 2011; Brown et al., 2013; Erickson et al., 2013; Hayes et al., 2013; Phillips et al., 2014; Svenson et al., 2014). Importantly, changes in brain volume have been identified as a potential mediator (Weinstein et al., 2012) by which exercise affects cognitive function in older adults. Humans display decreased cognitive function and reductions in brain volume as they age, which are most pronounced in prefrontal, temporal, and parietal gray matter (Good et al., 2001; Tisserand et al., 2004; Raz and Rodrigue, 2006) and are ameliorated by exercise (Colcombe et al., 2003, 2006; Bugg and Head, 2011; Erickson et al., 2011; Yuki et al., 2012). Thus, exercise is thought to be an important aspect of a healthy lifestyle that can help to preserve the neural substrates of cognition and promote healthy aging.

One shortcoming of the exercise neuroscience literature is that frequently, the words aerobic fitness (AF) and PA are used interchangeably; however, they represent different concepts that are measured in specific ways. Using these metrics interchangeably may be problematic with regards to how they are associated with morphological presentation of the brain. If, for example PA and AF measures indexed the same construct, we would expect no difference in neuroimaging assessments when using these measures interchangeably. Simply put, regional brain density would be predicted to be similar when comparing AF and PA measures. To attain an operational definition that could be referenced across studies, Caspersen and colleagues defined these frequently used terms as follows: PA is “any bodily movement produced by skeletal muscles that results in energy expenditure”, while physical fitness is a “set of attributes that people have or achieve and are either health- or skill-related” (Caspersen et al., 1985). Cardiorespiratory fitness or AF is the health-related component of physical fitness used most widely in brain aging research and it refers to the ability of the circulatory and respiratory systems to supply fuel during sustained PA and to eliminate metabolic byproducts that cause fatigue (Caspersen et al., 1985). The gold standard in AF measurement is maximal oxygen consumption (VO_2max_), which is usually obtained through a graded treadmill test. The majority of older persons (>75%), however, are unable to satisfactory complete a maximal graded exercise test (Hollenberg et al., 1998), which makes VO_2max_ utility in aging studies questionable. As such, studies have utilized sub-maximal estimated VO_2_ tests and/or function tests (12 min walk, for example), which are demonstrated to correlate with VO_2max_ (Peterson et al., 2003).

Many exercise in aging studies have found positive associations between objective AF measures and brain volumes/density (Gordon et al., 2008; Erickson et al., 2009, 2011; Ruscheweyh et al., 2011; Yuki et al., 2012). Others have assessed PA using self-report questionnaires asking participants about the length and frequency with which they perform certain leisure-time activities (Erickson et al., 2010; Flöel et al., 2010; Liang et al., 2010; Bugg and Head, 2011; Ho et al., 2011; Head et al., 2012); however self report is subject to recall biases and does not correlate highly with PA measured objectively via accelerometry (Troiano et al., 2008). Hence, not only do AF and PA represent different constructs, but whether PA is assessed objectively vs. subjectively can introduce measurement error and non-compatibility across studies.

Given the plethora of new studies attempting to identify the underlying mechanisms by which AF and PA improve brain health in aging, it is important to better characterize the relationship between brain health and different PA measuring tools. Although many studies investigating the relationship between AF/PA and gray matter density (GMd) have had somewhat overlapping results (Hayes et al., 2013), this is not the case for all studies (Honea et al., 2009). Similarly, if PA and AF indeed represent overlapping, but somewhat different constructs, associations between brain health and tools measuring these different constructs should differ.

Hence, the current study investigated the relationship between an objective AF measure (distance traveled on a 12 min treadmill test) and a self-report PA questionnaire (modified version of the Leisure Time Exercise Questionnaire) with GMd in cognitively normal community-dwelling older adults. We hypothesized that GMd associations would differ respective of AF level as compared to self-reported PA (srPA) given the nature of the different constructs and the objective and subjective nature of the indices, respectively. We also investigated whether AF or srPA modified the relationship between GMd and age and hypothesized that AF rather than srPA would modify this relationship given the objective, and thus more precise nature of this measure compared to srPA.

## Materials and methods

### Participants

Thirty cognitively healthy community-dwelling older adults participated in a study of aging and AF, srPA, functional MRI (fMRI), and structural MRI. fMRI findings for category fluency and finger tapping tasks related to aging and srPA have been reported elsewhere (McGregor et al., 2011; Zlatar et al., 2013). One participant was excluded from the current analyses due to evidence of ischemic event on MRI scan, thus 29 structural MRI scans were included in the current analyses. Participants were between the ages of 60 and 85 (mean = 68.38, SD = 5.99) and recruitment took place from flyers, advertisements, and ongoing aging research registries at the University of Florida in Gainesville. Years of education ranged between 12 and 20 (mean = 16.14, SD = 2.36). All participants were right-handed, native English speakers, who were deemed eligible for MRI scanning following an extensive screening protocol (e.g., no cardiac pacemaker, ferrous metal implants, or claustrophobia). Participants were free of a history of diagnosable neurological conditions (i.e., stroke, Alzheimer’s disease, Parkinson’s disease, mild cognitive impairment), history of head trauma with loss of consciousness, cardiac conditions, learning disabilities, attention deficit disorder, history of alcohol or drug abuse (for at least 6 months prior to participation), and psychiatric conditions. Older adults currently prescribed beta-blockers for hypertension management were not included in the study. All subjects obtained Mini-Mental State Examination (MMSE) scores ≥27 (Folstein et al., 1975) and scores on a comprehensive neuropsychological battery of tests were all within the average range of cognitive function, indicating no apparent global cognitive impairment at the time of testing. Cognitive testing took place during the 1st study session which was conducted 1 week prior to brain scanning (refer to Table 1 for a list of tests administered and corresponding scores). Signed informed consent was obtained from all participants according to guidelines established by the Health Science Center’s Institutional Review Board at the University of Florida. Participants were compensated for their participation in the study.

**Table 1:** **Participant characteristics and neuropsychological testing scores (*N* = 29)**.

	Mean	SD
Age	68.4	6.0
Education (years)	16.1	2.4
Physical activity (minutes per week)	242.9	204.8
Aerobic fitness (km)	0.89	0.34
Hopkins verbal learning test trial:1 (T score)	46.1	9.9
Hopkins verbal learning test total trials 1–3 (T score)	49.8	9.9
Hopkins verbal learning test delayed free recall (T score)	50.3	10.8
Hopkins verbal learning test delayed retention (T score)	50.9	15.4
Stroop color word reading (scaled score)	14.5	3.2
Trail making test part A (scaled score)	8.5	1.8
Trail making test part B (scaled score)	9.6	1.9
WAIS digit symbol (scaled score)	10.9	2.3
WAIS letter number sequencing (scaled score)	11.9	2.3
WAIS prorated working memory index (standard score)	108.1	11.8
Controlled oral word association test, letters FAS (scaled score)	10.3	2.1
Animal verbal fluency (scaled score)	10.3	2.5

*Notes: WAIS = Wechsler Adult Intelligence Scale, SD = Standard Deviation*.

### Aerobic fitness measurement

A modified Cooper AF test was conducted (Cooper, 1968) in which participants were asked to cover as much ground as possible by walking, jogging, or running on a treadmill for 12 min. The Cooper test is indicated as correlating with VO_2max_, with a demonstrated correlation coefficient of 0.92 when comparing it to a treadmill VO_2max_ test (Grant et al., 1995). Thus, distance traveled in 12 min (self-paced, variable speed) was used as the main AF variable for the current study. Twenty-nine participants completed the AF assessment with distance between 0.31 to 1.63 km covered in 12 min (mean = 0.89 km, SD = 0.34 km).

### Self-reported physical activity measurement

To measure self-reported PA level, a modified version of the Leisure-Time Exercise Questionnaire (LTEQ) was used, which is a 3-item scale that asks participants to rate how often they engaged in mild, moderate, and strenuous leisure-time exercise in a certain period of time (Godin and Shephard, 1985; Godin et al., 1986). The LTEQ is a reliable and valid measure of leisure-time PA behavior in adults (Jacobs et al., 1993) that allows for calculation of total number of minutes spent in light, moderate, and strenuous PA. In the present study, the total number of minutes spent in moderate and strenuous PA in one week was summed and used as the srPA variable. Twenty-nine participants completed the LTEQ questionnaire. The total amount of self-reported time spent performing moderate and strenuous PA ranged between 0 and 900 min in one week (mean = 242.86, SD = 204.78).

### Structural brain imaging parameters

Structural MRI scans for all participants were acquired on a three Tesla Achieva whole-body scanner (Philips), with an 8-channel SENSE radio frequency head coil, at the McKnight Brain Institute of the University of Florida. Structural TFE T1-weighted images were acquired for 160 × 1 mm sagittal slices (FOV = 240 mm; TE = 3.685 ms; TR = 8.057 ms; FA = 8 degrees; matrix = 256 × 256, voxel size = 1.0 mm × 0.938 mm × 0.938 mm).

### Procedure

An MRI screen was conducted via telephone to identify potential study candidates. Those who were MRI-eligible were asked to obtain written clearance from their primary care physician in order to participate in the treadmill test. The 12 min treadmill test took place during the 1st study session during which neuropsychological assessment was also conducted. Heart rate was monitored throughout the treadmill assessment. Prior to commencing the test, participants were asked to take some time to familiarize themselves with the treadmill and the test began once they reported verbally to the study staff that they were comfortable. After the treadmill test, participants were instructed on how to answer the modified LTEQ, which they completed daily for 7 days following the 1st study session. Structural brain imaging scans were obtained during the 2nd session following the 7 day srPA monitoring period.

### Brain image processing and statistical analyses

MRI structural images were analyzed using FSL-VBM, a voxel-based morphometry (VBM) analysis (Ashburner and Friston, 2000; Good et al., 2001), carried out with FMRIB Software Library (FSL; Oxford, UK) version 4.1 tools (Smith et al., 2004). This technique allows for the identification of different types of brain tissue (i.e., gray matter, white matter, and cerebrospinal fluid or CSF) at the voxel level by calculating the probability that each voxel contains a particular tissue-type. Pre-processing of the structural images for VBM analysis consisted of the following steps: (a) removing the skull and other non-brain tissue from the structural images using flsvbm_1_bet (Smith, 2002); (b) performing tissue-type segmentation to generate partial gray matter volumes for each participant in the native space using FAST4 (Zhang et al., 2001); and (c) aligning the resulting gray matter partial volumes to MNI152 2 mm standard space using the non-linear registration tool FNIRT (Andersson et al., 2007a, b), which uses a b-spline representation of the registration warp field (Rueckert et al., 1999). The resulting images were concatenated and averaged to create a study-specific template containing the registered gray matter images for each of the 29 older adult subjects (fslvbm_2_template-n). All of the gray matter images were then non-linearly registered to the study-specific template (fslvbm_3_proc). To account for changes in voxel size that occur within the image registration process (expansion and contraction), all registered partial volume images were modulated by dividing by the Jacobian of the warp field (fslvbm_3_proc). The modulated, segmented images were then smoothed with an isotropic Gaussian kernel of sigma 2 mm, which corresponds to 4.6 mm full-width half-max (FWHM).

We used the randomize option in FSL, which is a permutation-based program allowing for modeling and inference testing using standard general linear model design, when the null distribution of a statistic map is unknown. To describe the unique relationship between GMd, age, fitness level, and srPA, we conducted two voxel-wise multiple regression models under the general linear model, higher level/non-time series design option in FSL: (1) The first regression model used voxel-wise GMd as the dependent variable and age, AF (distance traveled in 12 min during treadmill test), and the interaction term between age and AF as independent variables; and (2) The second regression model included voxel-wise GMd as the dependent variable and age, srPA (self-reported minutes of moderate + strenuous activity in 7 days), and the interaction term between age and srPA as independent variables. These two whole-brain analyses were conducted using data from all 29 participants, and the independent variables were centered (demeaned) and continuous. The resulting probability maps were corrected for family-wise error (*p* < 0.05) using the Threshold-Free Cluster Enhancement (TFCE) method. This method enhances cluster-like regions more than background (noise) by creating output that is a weighted sum of all of the local clustered signal, without the need for arbitrary cluster thresholding. If the voxel-wise analyses returned any significant interaction terms (age*AF or age*srPA), the average per-subject GMd values were extracted from significant clusters and further analyzed using IBM SPSS (version 21) to characterize the direction of the interaction.

Furthermore, given the reported associations between GMd and the hippocampus, anterior cingulate cortex, lateral frontal, and lateral parietal regions (for a recent review, refer to Hayes et al., 2013), a *post hoc* region of interest (ROI) analysis was conducted using anatomically-defined ROIs based on the cortical and subcortical Harvard-Oxford atlases available in FSL. Pearson bivariate correlations were conducted between mean GMd values extracted from each ROI and AF and srPA using IBM SPSS. ROIs included: right and left hippocampi, inferior and middle frontal gyri, frontal pole, anterior cingulate cortex, and the angular and supramarginal gyri.

## Results

### Overlap between aerobic fitness and self-reported physical activity on gray matter density

**Figure 1:**
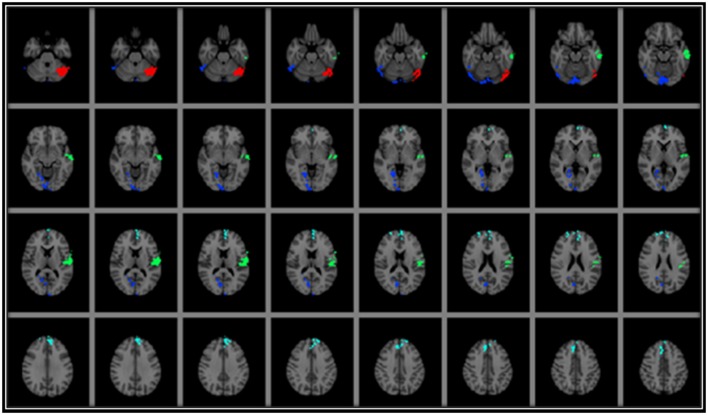
**Results from voxel-wise regression models displaying the brain regions in which GMd was associated with AF and with srPA after correcting for age**. As can be seen, there was no overlap between GMd in brain regions associated with AF and those associated with srPA. Areas where there was a significant association between srPA and GMd included: (1) bilateral occipital poles, lingual and left fusiform gyri, and right calcarine cortex (occipito-temporal cluster—BLUE); (2) left central opercular cortex and middle and superior temporal gyri (left perisylvian cluster—GREEN); and (3) bilateral frontal poles, superior frontal gyrus, and paracingulate cortex (frontal cluster—CYAN). There was a significant association between AF and GMd in the left cerebellum (RED) Clusters are overlaid on the MNI 2 mm brain template. GMd = Gray matter density. Left = right; Right = left.

Based on the voxel-wise analyses described above, there was no overlap in regional GMd between the areas significantly associated with AF and those significantly associated with srPA (Figure 1), suggesting that AF and srPA are associated with GMd in distinct and non-overlapping brain regions. The bivariate correlation between AF and srPA measures was *r* = 0.43, *p* = 0.02.

### Relationship between gray matter density and self-reported physical activity

**Figure 2:**
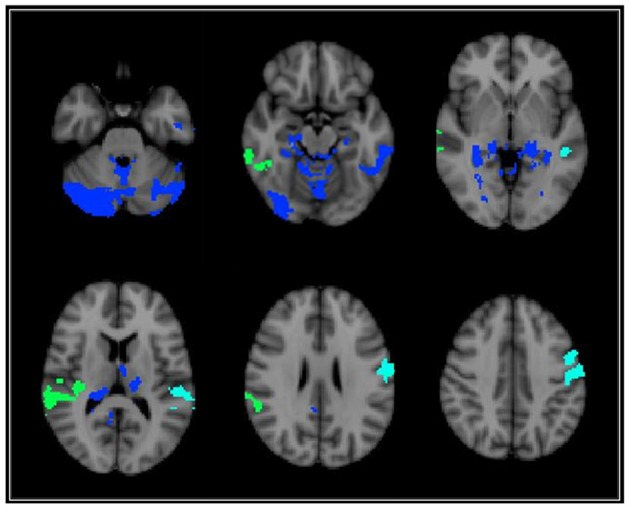
**Brain regions depicting areas where there was a significant voxel-wise interaction between age and AF on GMd**. These three significant interaction clusters were located on the: (1) bilateral cerebellum, lingual, and fusiform gyri, and left inferior and middle temporal gyri [temporo-cerebellar cluster—BLUE]; (2) right superior, middle and inferior temporal gyri, right supramarginal gyrus and planum temporale (right perisylvian cluster—GREEN); (3) and left precentral and postcentral gyri (left superior frontal cluster—CYAN). (See Table 2 for location coordinates and regression coefficients). Clusters are overlaid on the MNI 2 mm brain template. GMd = Gray matter density. Left = right; Right = left.

Voxel-wise analyses indicated that srPA was significantly associated with GMd in three discrete clusters after correcting for age: (1) bilateral occipital poles, lingual and left fusiform gyri, and right calcarine cortex (occipito-temporal cluster depicted in BLUE on Figure 1); (2) left central opercular cortex and middle and superior temporal gyri (left perisylvian cluster depicted in GREEN on Figure 1); and (3) bilateral frontal poles, superior frontal gyrus, and paracingulate cortex (frontal cluster depicted in CYAN on Figure 1). The interaction between srPA and age on GMd was not statistically significant.

### Relationship between gray matter density and aerobic fitness

Voxel-wise analyses indicated that AF was significantly associated with GMd after correcting for age in one cluster located in the left cerebellum (depicted in RED on Figure 1) after family-wise error correction (TFCE *p* < 0.05). The interaction term between age and AF was significantly associated with GMd in three clusters: (1) bilateral cerebellum, lingual, and fusiform gyri, and left inferior and middle temporal gyri (temporo-cerebellar cluster depicted in BLUE on Figure 2); (2) right superior, middle and inferior temporal gyri, right supramarginal gyrus and planum temporale (right perisylvian cluster depicted in GREEN on Figure 2); and (3) left precentral and postcentral gyri (left superior frontal cluster depicted in CYAN on Figure 2).

**Table 2:** **Coefficients for the interaction term between age and fitness level on GMd in the three significant clusters from voxel-wise regression**.

			Lower fitness		Higher fitness	
Cluster location	Voxels	COG X, Y, Z	β	***t***	**β**	***t***
Left superior frontal	1146	73.2, 56.2, 49.9	−0.86^**^	−4.04	0.32	1.08
Right perisylvian	1318	17.8, 46.9, 39.8	−0.88^**^	−4.12	0.23	0.77
Temporo-cerebellar	10718	43.8, 32.7, 23	−0.6^*^	−2.65	0.73^*^	2.3

Notes: GMd = Gray matter density; AF = Aerobic fitness; COG = Center of gravity in MNI 2 mm coordinates; β = standardized beta coefficient; t = t-statistic. Statistically significant coefficient **p < 0.01, *p < 0.05. βs represent the extent and direction of the relationship between age and GMd by AF level for each significant interaction cluster (age*AF on GMd). As can be seen, for those with lower AF, greater age is associated with lower GMd in each of the significant brain regions; whereas greater age is associated with higher GMd in temporo-cerebellar regions for those with higher AF levels.

**Figure 3:**
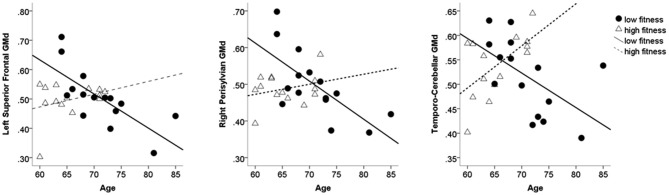
**Scatter plots depicting individual data points for the three clusters where the interaction term between AF and age on GMd was significant based on the voxel-wise regression model**. Table 2 shows the regression coefficients for the moderating effect of AF on age-related GMd changes. AF = Aerobic fitness; GMd = Gray matter density.

To decompose the interaction term between age and AF on GMd in these significant clusters, a mask of these clusters was created and the averaged GMd for each participant extracted to conduct further analyses using IBM SPSS. Hierarchical linear regression models were conducted with the averaged GMd for each significant cluster as the dependent variable, and age, AF (dichotomized using a median split), and the interaction term between age and AF as independent variables. It is important to note that AF was dichotomized into high and low fitness groups using a median split in order to characterize the interaction term and not for the purposes of comparing low and high AF groups. For those with lower AF, there were significant negative associations between age and GMd in these three regions, while for individuals with higher fitness, there was a significant positive association between age and GMd in temporo-cerebellar regions. See Table 2 for regression coefficients and Figure 3 for scatter plots depicting the interaction between age and AF on GMd in the three significant clusters from the voxel-wise regression analyses.

### *Post hoc* ROI analyses

There were no significant associations between the AF measure and GMd in any of the ROIs (anterior cingulate cortex, inferior and middle frontal gyri, frontal pole, angular and supramarginal gyri, and hippocampi). srPA was associated with GMd in the left hippocampus only (*r* = −0.403, *p* = 0.03, *N* = 29).

## Discussion

Many studies have identified positive associations between PA or AF and different measures of brain health; however the constructs being measured and the tools used to measure them have varied between studies, predicting differential outcomes when it comes to brain morphology. The main purpose of the current study was to evaluate if objectively-measured AF and srPA levels had differential associations with GMd in cognitively healthy older adults and to investigate if the relationship between age and GMd would vary as a function of AF or srPA.

We found that objectively-measured AF was associated with increased GMd in the left cerebellum, while srPA was correlated with increased GMd in occipito-temporal, left perisylvian, and frontal regions. These results suggest that GMd in brain regions associated with AF and srPA levels vary depending on which measure was used to assess them. More importantly, AF level was a significant moderator of the relationship between age and GMd, while the same was not true of srPA. For those with lower AF levels, there were strong negative associations between age and GMd in left superior frontal and right perisylvian regions suggesting that, as age increases, GMd decreases in this group. To the contrary, individuals with higher AF showed a positive association between GMd and age in temporo-cerebellar regions, indicative of higher GMd in this region as a function of higher age. These results indicate that objectively-measured AF level, but not srPA, may serve as a buffer to delay or reduce age-related decreases in GMd. This is consistent with previous studies showing that cardiorespiratory fitness is associated with sparing of gray matter atrophy in areas that are selectively affected by the aging process (Colcombe et al., 2003). Alternatively, declines in AF may have accelerated age-related GM atrophy; however, whether lower AF causes accelerated atrophy or whether higher AF mitigates age-related GM atrophy cannot be ascertained given the cross-sectional nature of the current study. Future studies that follow both fit and unfit individuals over time could help to answer this question.

Although the purpose of the current study was to find voxel-wise associations between AF and srPA with GMd, we conducted *post hoc* ROI analyses to determine if these two measures were correlated to GMd in brain regions previously identified to be sensitive to exercise and PA in older adults. ROI analyses indicated that there was no association between the objective AF measure and GMd in areas previously shown to be associated with cardiorespiratory fitness such as the hippocampus, anterior cingulate cortex, frontal and parietal regions. This may be due to the fact that we currently used a modified version of the Cooper test, while many other studies have employed VO_2max_ measurement (Colcombe et al., 2003, 2006; Gordon et al., 2008). While AF was not associated to GMd in these ROIs, there was a negative association between srPA and GMd in the left hippocampus, indicating that those with higher self-reported levels of PA had lower GMd in the left hippocampus. Although this seems counter intuitive, it is not surprising given the self-report nature of this measure, which makes it unreliable. Moreover, in the current study, srPA failed to moderate the effects of age on GMd as was the case for the AF measure.

Two factors may affect differences between AF and srPA in their associations with GMd and age. The first is that even though AF and PA are statistically related (as in the current study), they represent different constructs. Hence, it is not surprising that they predict differential brain morphology when the variance for age within this older sample is removed. The second factor is that our measure of AF was objective and our measure of PA was self-report. The lower reliability (and, therefore, greater error variance) of self-report measures may account for the lack modulation of age-related loss in GMd by PA. It is critical to know whether PA can modulate age-related GMd loss. Hence, future studies investigating these associations should rely on objective measurement of PA since srPA is frequently overestimated (Troiano et al., 2008). PA levels can be reliably measured using research-grade accelerometers (Troiano et al., 2008), which provide a summary of time spent in sedentary, light, moderate, and vigorous PA behaviors, allowing for the study of associations between cognitive function and brain health with different PA intensity levels. Given the recent associations between accelerometer-measured sedentary time with brain and physical health in older adults (Gennuso et al., 2013; Zlatar et al., 2014), studying the independent effects of sedentary time on brain and cognitive health will become an important goal for future studies.

The present study is limited by a small sample size and by the fact that the *post hoc* regression analyses were not conducted by an investigator blinded to the AF data for each participant. Moreover, these results must be interpreted within the context of the current sample, which may not be representative of the general older adult population given their high education level and exclusion of those taking beta blockers to control hypertension (which is a condition extremely common in aging). Similarly, joint pain, which is common in the elderly and affects the ability to stand or walk (Keenan et al., 2006), may have limited the distance participants were able to cover during the treadmill test. Future studies should take this into account to control for its effects on fitness and PA testing.

In conclusion, the current study found no overlap in regional GMd associated with objectively-measured AF and a self-report measure of PA in cognitively unimpaired older adults, highlighting the need to better understand the associations between brain outcomes and different measures of AF and PA. We also found that the relationship between GMd and age changed as a function of AF, but not srPA, attesting to the neuroprotective role of AF in aging by sparing GMd loss as age increases. Future studies investigating the underlying mechanisms by which exercise improves brain health should use objective measurement of PA and AF that is comparable across studies.

## Author and contributions

All authors contributed substantially with conception and study design, data acquisition and analyses, data interpretation, manuscript drafting and revisions, and manuscript approval.

## Conflict of interest statement

The authors declare that the research was conducted in the absence of any commercial or financial relationships that could be construed as a potential conflict of interest.
